# Reinforcement Contingency Learning in Children with ADHD: Back to the Basics of Behavior Therapy

**DOI:** 10.1007/s10802-019-00572-z

**Published:** 2019-07-11

**Authors:** Hasse De Meyer, Tom Beckers, Gail Tripp, Saskia van der Oord

**Affiliations:** 1 Research Unit Behavior, Health and Psychopathology, KU Leuven, Tiensestraat 102 box 3720, 3000 Leuven, Belgium; 2 Leuven Brain Institute, KU Leuven, Leuven, Belgium; 3 Human Developmental Neurobiology Unit, Okinawa Institute of Science and Technology Graduate University, 1919-1 Tancha, Onna, Kunigami District, Okinawa, Prefecture 904-0412 Japan; 4 Developmental Psychology, University of Amsterdam, Nieuwe Achtergracht 129, Amsterdam, 1018 WS The Netherlands

**Keywords:** Attention deficit hyperactivity disorder, Partial reinforcement extinction effect, Operant learning, Treatment

## Abstract

Reinforcement deficits in ADHD may affect basic operant learning processes relevant for Behavioral Treatment. Behavior acquired under partial reinforcement extinguishes less readily after the discontinuation of reinforcement than behavior acquired under continuous reinforcement, a phenomenon known as the Partial Reinforcement Extinction Effect [PREE], which has great relevance for the emergence of behavioral persistence. The present study examined acquisition and extinction of operant responding under partial and continuous reinforcement in children with and without ADHD. In addition, we evaluated the effectiveness of gradual stretching the reinforcement rate during acquisition for remedying potential acquisition or extinction deficits under partial reinforcement in ADHD. In an operant learning task designed to mimic the task confronted by an animal in a Skinner box, 62 typically developing and 49 children with ADHD (age: 8–12) were presented with a continuous, partial or gradually stretching reinforcement scheme followed by extinction. Both groups of children acquired the instrumental response more slowly and exhibited more behavioral persistence (reduced extinction) when responding was initially reinforced under partial relative to continuous reinforcement, with no differences between groups. Progressive ratio stretching resulted in faster acquisition than partial reinforcement yet promoted equal behavioral persistence, again without differences between ADHD and TD groups. Unlike suggested by previous research, children with ADHD exhibit neither an acquisition deficit under partial reinforcement nor a deficit in PREE. Of relevance for Behavioral Treatment, gradual reinforcement stretching can be used to facilitate response acquisition over purely partial reinforcement while maintaining equal behavioral persistence upon reward discontinuation.

**Electronic supplementary material:**

The online version of this article (10.1007/s10802-019-00572-z) contains supplementary material, which is available to authorized users.

## Background

Children with Attention Deficit Hyperactivity Disorder [ADHD] display elevated levels of inattentive, hyperactive and impulsive behaviors, which are inconsistent with their developmental level (American Psychiatric Association 2013). The disorder, typically diagnosed in early to middle childhood, is common (5–7%) (Polanczyk et al. 2007) and leads to impairment in multiple areas of functioning (Wehmeier et al. 2010). It is also associated with long-term risk for adverse outcomes in later life (American Psychiatric Association 2013; Wehmeier et al. 2010). While the specific etiology of ADHD remains uncertain it is known to be highly heritable (Biederman and Faraone 2005).

In most countries, Behavioral Parent Training [BPT] is recommended as a first line, non-pharmacological treatment for children with mild to moderate ADHD (Daley et al. 2018; B. K. Schultz et al. 2017). While the effectiveness of BPT for comorbid oppositional symptoms and for improving children’s emotional, social and academic functioning is well established, it is less effective than pharmacotherapy in reducing the core symptoms of ADHD (Daley et al. 2014; Van der Oord et al. 2008). Moreover, it has limited long-term effects (Lee et al. 2012). Behavioral treatment is strongly embedded in operant learning principles; i.e., adaptive behavior is reinforced, and non-adaptive behavior is ignored or punished. However, theoretical models and empirical research suggest that these operant learning processes might be disrupted in children with ADHD (Sagvolden et al. 2005; Tripp and Wickens 2008). Adapting parenting programs in view of basic deficits in learning processes may enhance the (long-term) effectiveness of our current behavioral treatments in managing ADHD symptoms (Sonuga-Barke and Halperin 2010).

Surprisingly, although closely linked to principles of BPT, deficits in ADHD in reinforcement learning have received far less research attention than motivational deficits. Constructs such as delay aversion (the tendency of children with ADHD to escape or avoid delay of gratification) and delay discounting (preferring a smaller, earlier reward over a larger but delayed reward) are considered to have their cause in basic reinforcement-learning deficits, and have been the focus of several theoretical accounts of ADHD (Sonuga-Barke 2003). These motivational deficits in ADHD are widely studied experimentally, both at the behavioral and neural level (Luman et al. 2005, 2015), but the core mechanisms subserving these constructs, i.e., reinforcement processes and instrumental learning, have received only limited attention.

Two prominent theoretical accounts do focus on altered reinforcement learning as a primary deficit in ADHD (Sagvolden et al. 2005; Tripp and Wickens 2008). According to the Dynamic Developmental Theory, symptoms of ADHD originate from a diminished dopamine signal in anticipation of or following a reinforcer, which causes a steep temporal discounting slope, prompting a preference for a smaller but immediate over a larger but delayed reward (Sagvolden et al. 2005). Especially when reinforcement is delayed, the fast attenuation of the dopamine response (or reward signal at the neural level) prevents the formation of a strong association between conditioned stimuli or instrumental responses and reinforcement, leading, for instance, to symptoms of inattention. Tripp and Wickens (2008) proposed a related theory of hypo-dopaminergic functioning in children with ADHD. According to their Dopamine Transfer Deficit theory, in children with ADHD the firing of dopamine cells in response to unexpected reward fails to fully transfer from the actual reward to stimuli or responses that reliably predict reward delivery. Therefore, in children with ADHD, in the case of delayed or discontinuous reinforcement, no anticipatory dopamine signal bridges the delay between the stimuli that predict the reward and the actual reward, resulting in diminished learning as compared to Typically Developing [TD] children. However, when reward is immediate and continuous there is no disruption of dopamine signaling, which should result in a learning curve indistinguishable from that of TD children (Tripp and Wickens 2008).

A small number of early experimental studies confirm deficits in reinforcement learning (acquisition: the establishment of an instrumental conditioned response) under partial reinforcement but not under continuous reinforcement, in children with ADHD compared to TD children (Douglas and Parry 1994; Freibergs and Douglas 1969; Parry and Douglas 1983); but see (Cunningham and Knights 1978). However, the conclusions that can be drawn from this research are limited, due to differences between studies in the type of reinforcement used (e.g., monetary reward, tokens or presents), the magnitude of reinforcement used (e.g., earning 4 dollars, a prize of 25 cents, etc.) (Carlson and Tamm 2000; Douglas and Parry 1994; Luman et al. 2005; W. Schultz 2000) and a lack of true partial reinforcement conditions (e.g., the researchers provided verbal feedback about response accuracy on “non-reinforced” trials which might also be considered a reinforcer) (Luman et al. 2005; Parry and Douglas 1983). For the study of Parry and Douglas (1983) it is unclear whether a differential outcomes procedure was used, which could have distinctive effects on learning (Nevin et al. 2009; Parry and Douglas 1983; Pelham et al. 1986).

Moreover, in the studies mentioned above, differences between continuous and partial reinforcement were assessed using concept learning (Freibergs and Douglas 1969), delayed reaction time (Parry and Douglas 1983), and spelling tasks (Pelham et al. 1986). These tasks involve cognitive learning strategies such as conceptual processing or reversal learning and are most likely correlated with intelligence. As a result, they are only partly focused on core learning processes per se. In other words, group differences in these earlier studies, with the relatively complex tasks that they typically used, might be due to differences in attentional or conceptual skills rather than reflecting differences in elementary reinforcement learning (Freibergs and Douglas 1969; Segers et al. 2018).

Despite its importance for the understanding of behavioral persistence, there are even fewer empirical studies assessing the effects of the discontinuation of reinforcement (extinction) on responding in children with ADHD (Ayllon et al. 1975; Worland et al. 1973). Behavior that was acquired under conditions of partial reinforcement (i.e., using a less than 100% contingency between behavior and reinforcement) is more persistent under extinction than behavior that was acquired under continuous reinforcement. This phenomenon, tested mainly in animal studies, is known as the Partial Reinforcement Extinction Effect (PREE; Humphreys 1939). Interestingly, the two reinforcement-centered theoretical accounts of ADHD, Sagvolden’s (2005) Dynamic Developmental theory and Tripp and Wickens’ (2008) Dopamine Transfer Deficit theory, make opposite predictions regarding the behavior of children with ADHD under extinction. According to Tripp and Wickens (2008), due to hypo-efficient dopamine transfer, extinction should generally occur faster in children with ADHD relative to TD children. This faster extinction may restrict the occurrence of a PREE and thereby the emergence of generalized behavioral persistence in ADHD (Taddonio and Levine 1975). According to Sagvolden’s (2005) theory, extinction of previously reinforced behavior should generally be slower in ADHD, with a supposed deficit in extinction in children with ADHD leading to increased behavioral variability and bursts of hyperactive behavior. Despite these different predictions regarding extinction, the two accounts converge in proposing impaired acquisition of behavior under conditions of partial reinforcement in ADHD.

In contrast to the extensive animal research (Sangha et al. 2003), evidence for the PREE from human studies is less clear-cut (Flora and Pavlik 1990; Hochman and Erev 2013) and research with children is very limited (for exceptions, see Pittenger 2002; Segers et al. 2018). The hypothesized deficits in partial reinforcement learning and extinction in ADHD would potentially lead to poorer acquisition of new or adaptive behavior together with less persistence of newly learned behavior over time leading to diminished generalized behavioral perseverance (Tripp and Wickens 2008). To overcome these PREE deficits and improve generalized adaptive behavior in ADHD, strategies to enhance behavioral persistence need to be explored.

Animal research indicates that one way to overcome a partial reinforcement deficit while preserving behavioral persistence under extinction, is a procedure known as stretching the ratios, i.e., gradually weaning the density of reinforcement during acquisition from very high levels (100%) to very sparse levels of reward (e.g., 20%). To our knowledge, only one study has explored the potential effectiveness of stretching the ratios in children with ADHD. A study with hyperactive boys showed that a shift from a variable 1-min interval (VI 1) reinforcement schedule to a more sparse schedule (VI 3) was more effective in increasing on-task behavior when a variable 1.5-min (VI 1.5) was interjected (Barkley et al. 1980). However, no comparison of the effect of stretching the ratios on the behavior of children with ADHD and TD children was made.

In sum, despite the central role of impaired reinforcement learning in theoretical models of ADHD (Luman et al. 2010), there is a lack of reliable evidence regarding elementary reinforcement learning under continuous and partial reinforcement in children with ADHD, its sensitivity to extinction and PREE, and potential methods to overcome hypothesized deficits by stretching the ratios. The present study therefore examined instrumental reinforcement learning (acquisition and extinction) in children with ADHD and TD children under partial and continuous reinforcement schedules, using a relatively simple task closely modeled on procedures used in operant learning experiments in animals (i.e., closely resembling a Skinnerian instrumental learning task). In this touchscreen computer task, participants were required to learn which of ten differently colored balls, presented together on a computer screen, was the correct one. In acquisition, participants were reinforced for correct responses under different reinforcement schedules, in a between-subjects design (either continuous, partial or stretching the ratios), followed by an extinction phase in which no reinforcements were delivered irrespective of performance on the task.

We expected that TD children as well as children with ADHD would acquire the stimulus-response association faster in the continuous reinforcement condition than in the stretching the ratios condition, with those in the partial reinforcement condition expected to learn most slowly. While acquisition of the correct response was expected to proceed more quickly under continuous reinforcement, we expected the learned behavior to extinguish faster following continuous reinforcement than when behavior was acquired under stretching-the-ratios or partial reinforcement conditions. Between the latter two conditions, we did not expect significant differences in the speed of extinction.

With regard to between-group (ADHD vs TD) differences during acquisition, based on previous findings regarding the performance of children with and without ADHD (Freibergs and Douglas 1969; Parry and Douglas 1983), we expected to find a difference between the groups under partial but not under continuous reinforcement. With regard to the effects of extinction on the behavior of children with and without ADHD, we measured the number of correct responses (the outcome measure for a PREE) as well as the degree of exploratory behavior (i.e., the total number of responses during extinction), which can be considered an adaptive response when reinforcement is no longer provided for previously rewarded behavior, but without strong a-priori predictions. According to the Dynamic Developmental theory (Sagvolden et al. 2005), extinction of newly learned behavior should be slower in children with ADHD relative to TD children. However, according to the Dopamine Transfer Deficit hypothesis (Tripp and Wickens 2008), extinction should generally occur faster, restricting the occurrence of a PREE and possibly the emergence of generalized behavioral persistence in ADHD. However, neither model makes predictions about the impact of different reinforcement schedules during acquisition on subsequent responding under conditions of extinction. Therefore, we expected to observe a main effect of group but no interaction effects on either outcome measure during extinction. Finally, although assumed by researchers and clinicians to be beneficial to remediate expected deficits in partial reinforcement learning in children with ADHD (Parry and Douglas 1983), the effects of stretching the ratio of reinforcement have not been widely examined in humans, let alone in children with ADHD (but see Barkley et al. 1980). We expected that stretching the ratios would alleviate the anticipated partial reinforcement impairment in acquisition in children with ADHD.

## Method

### Participants

A total of 119 children, aged 8 to 12 years, participated in the study. Sixty-four typically developing children were recruited from regular schools and 55 children with a prior diagnosis of ADHD (any type) were recruited through the clinical networks of the authors. The diagnostic status of the clinical group was confirmed by the Disruptive Behavior Disorders section of the Diagnostic Interview Schedule for Children, Parent Version^1^ (PDISC; Shaffer et al. 2000) or the Attention Deficit Hyperactivity section and Behavioral Disorders Supplement of the Schedule for Affective Disorders and Schizophrenia for School-Age Children-Present and Lifetime version^2^ (K-SADS; Kaufman et al. 1997), depending on the testing location. All participating children met the following inclusion criteria:

*For both groups*: (a) an estimated full IQ score ≥ 80, established with the short version of the Dutch version of the Wechsler Intelligence Scale for Children (WISC-III-NL; Kort et al. 2005),^3^ (b) absence of a clinical diagnosis of Autism Spectrum Disorder (ASD) or any sensory or motor impairment or neurological disorder as reported by the parents, (c) not being prescribed any psychotropic medication other than methylphenidate or dexamphetamine.

*For children with ADHD*: (a) presence of a prior clinical DSM-IV (American Psychiatric Association 2013) diagnosis of ADHD of any subtype, established by a certified psychologist or psychiatrist and confirmed by the PDISC (Shaffer et al. 2000) or K-SADS (Kaufman et al. 1997), (b) absence of a clinical diagnosis of Conduct Disorder (CD) as measured by the CD section of the PDISC/K-SADS, and (c) willingness to discontinue stimulant medication use for 24 h prior to testing (Greenhill 1998).

*For TD children*: (a) absence of a (sub)clinical score (95.5th to 100th percentile) on the ADHD scales of the Dutch version of the Disruptive Behavior Disorder Rating Scale (DBDRS; Dutch translation: Oosterlaan et al. 2008), (b) absence of a (sub)clinical score (97.7th to 100th percentile) on the CD scale of the DBDRS (Oosterlaan et al. 2008).

### Inclusion Measures

*WISC-III-NL, short version*: To estimate IQ, two subtests from the Dutch version of the WISC-III (Block Design and Vocabulary) were administered. This two-subtest form is strongly correlated with the full-scale IQ and exhibits satisfactory reliability and validity (.91 and .86) (Sattler 2001).

*Structured interview:* Parents were administered either the Diagnostic Interview Schedule for Children, Parent Version (PDISC; Shaffer et al. 2000) or the Schedule for Affective Disorders and Schizophrenia for School-Age Children-Present and Lifetime version (K-SADS; Kaufman et al. 1997). Both interviews use DSM-IV criteria to assess ADHD, CD and ODD, with satisfactory psychometric properties (﻿﻿test-retest reliability of .63 for KSADS-PL and .79 for PDISC) (Kaufman et al. 1997; Shaffer et al. 2000). Interviews were carried out by students working toward their Master’s degree in clinical psychology, or licensed clinical psychologists, all trained to administer the interviews by the first author.

*DBDRS*: The Dutch version of the Disruptive Behavior Disorder Rating Scale (Oosterlaan et al. 2008) is a 42-item questionnaire designed to be completed by parents of children between 6 and 16 years of age. It includes four DSM-IV-TR based scales measuring Attention, Hyperactivity/Impulsivity, Oppositional Defiant Disorder (ODD) and Conduct Disorder (CD). The internal consistency of the scales in a Flemish sample is good for Attention (α = .90), Hyperactivity/Impulsivity (α = .87) and ODD (α = .88) and acceptable for CD (α = .66) (Oosterlaan et al. 2008).

### Reinforcement Learning Task

**Fig. 1:**
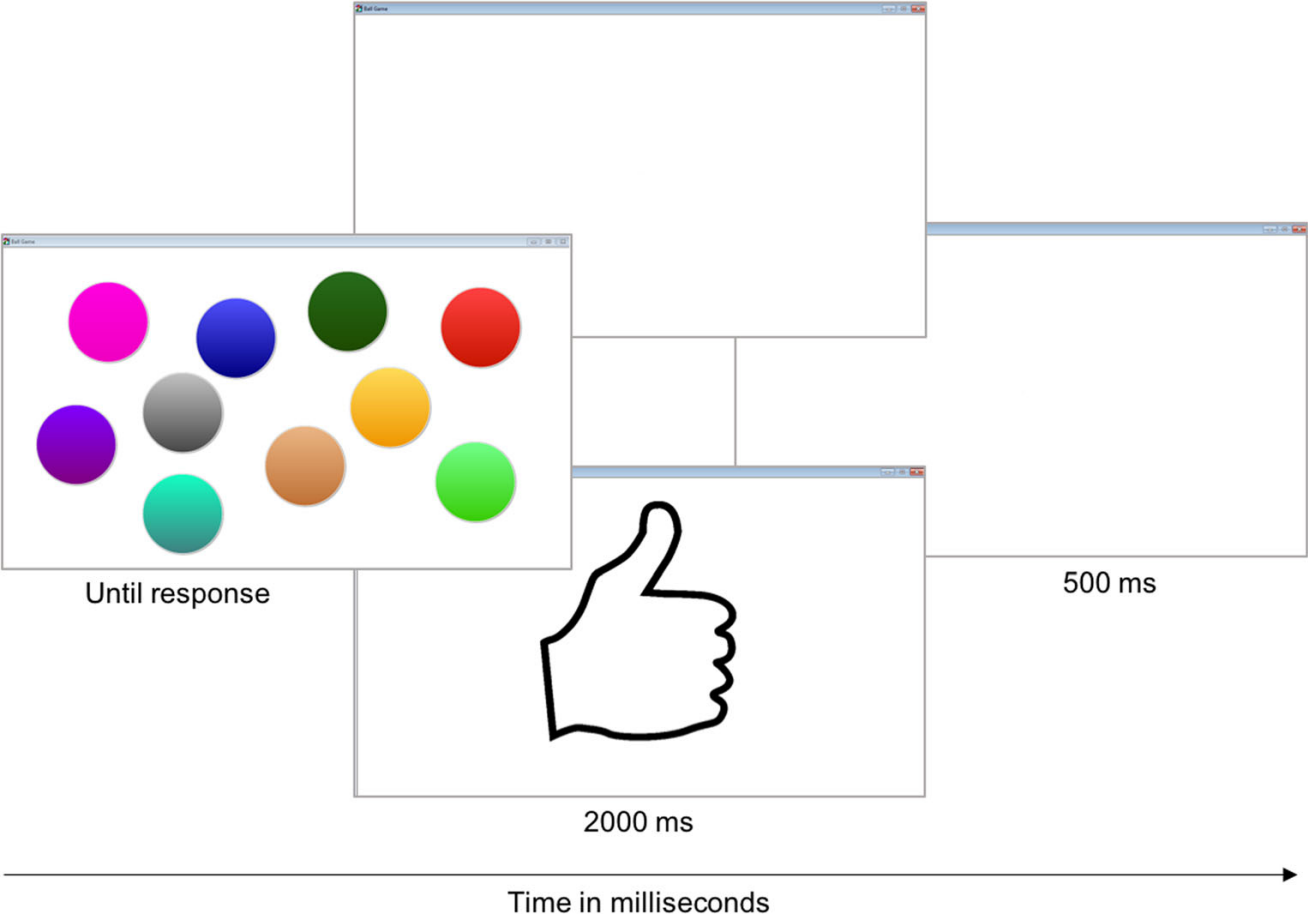
Time course of the operant learning task “Ball game”. Children are presented with a screen and ten colored circles which they can press. After pressing the correct one, a thumbs up appears followed by an inter-trial interval of 500 ms. When an incorrect response is made (wrong ball) or when the child is not scheduled to receive reinforcement, a white screen appears followed by an inter-trial interval. After the inter-trial interval, the circles are randomly rearranged. For a video of the task see: https://osf.io/ztq6y/?view_only=883a1dfa89684769a4918df652358045

The ball game is a newly developed, free-operant instrumental learning task. The task was designed to present children with a situation similar to that faced by a rat learning to obtain reinforcement (food) by pressing a lever in a skinner box. The ball game includes an acquisition phase and an extinction phase, presented on a computer touchscreen (Lenovo, Windows 8, 15-in.). On the screen, ten differently colored circles (“balls”) are presented in random locations (see Fig. 1). Each ball is clearly distinguishable in color from the others; they are not designed to serve as distractors. After every response, the location of the colored circles changes randomly. The children receive the simple instruction to find out which of the colored balls is the correct one by pressing them.

During the acquisition phase, pressing the correct ball is reinforced with a “thumbs up” presented on the screen (secondary reinforcer) followed by the experimenter giving the child an M&M candy (or Skittles if they disliked M&M) (primary reinforcer); the child is instructed to eat the candy as soon as they receive it. Satiation was not observed, i.e., the children continued to want to eat the candy as soon as they received it. The child is not informed of the number of reinforcers available (candies are hidden from sight) or the reinforcement schedule. Pressing any of the other balls results in a white screen followed by a random rearrangement of the balls on the screen. Irrespective of the result of the touchscreen response (reinforcement or white screen), after 500 ms the colored balls reappear on the screen in a random distribution. The color of the “correct” ball is randomly selected for each participant.

Participating children were randomly assigned to one of three different reinforcement conditions (between-subjects): continuous reinforcement, partial reinforcement, and stretching the ratios. Under continuous reinforcement (100% reinforcement; CRF) every touchscreen press on the correct colored ball is reinforced by both a primary and secondary reward. Under partial reinforcement, only 1 in 5 correct touchscreen responses is rewarded using a variable ratio schedule where, on average, every 5th correct response is reinforced (20% reinforcement; PRF) (both primary and secondary). Under stretching the ratios, the probability of reinforcement (both primary and secondary) for correct responses gradually decreases from continuous to sparse (100% – 50% – 33% – 25% – 20%) and from fixed (reinforcement is provided after a fixed number of trials, e.g., FR2: every 2nd trial) to variable (reinforcement is provided after a variable number of correct trials, e.g., VR4: on average every 4th trial), as follows: FR1 – FR2 – FR3 – VR4 – VR5. Once 20 rewards are received, irrespective of the reinforcement condition, a 2-min extinction phase is implemented. In this phase all responses trigger a white screen irrespective of their accuracy followed by rearrangement of the balls.

The approximate task duration ranged between 5 and 30 min, depending on the reinforcement schedule and the participant’s speed of learning. The primary measure of reinforcement learning (acquisition) is the total number of trials needed to earn 20 rewards (both correct and non-correct trials), counting from the first trial on which a child is reinforced. The primary measures of extinction are (a) *behavioral persistence* of the previously acquired response, measured by the number of responses to the previously rewarded ball during the 2-min extinction period and (b) *exploratory behavior* after reinforcement termination, measured by the total number of responses to any of the balls during the 2-min extinction period.

### Design and Procedure

Children and parents were informed about the study, and what it involved, through separate child and adult information letters. After obtaining their oral (children) and written consent, parents completed the structured interview (clinical sample only) and filled out the questionnaires (demographic questionnaire and DBDRS). After confirming that children met the inclusion criteria, they were randomly assigned to one of the three conditions (described below) with stratification for age, gender and the presence of ADHD, using a between-subjects design. Due to practical and recruitment constraints, the task was administered either during the first or last test session of a larger study in which there were multiple test sessions (100 min/session). Within this test session, the ball game was always the last of a series of different tasks. Of all participating children, 20% completed this task in the first test session and 80% in the last test session. There were no demographic or performance differences between these two groups.

Children sat in front of a touchscreen computer in a quiet distraction-free room. Each child was given the following instructions: “In a minute you will play a game, pay close attention because no further instructions will be given”. The task instructions appeared on the computer screen and were read out loud by the experimenter and the child was instructed to read along in silence: “In a moment 10 colored balls will appear on the screen. By pressing the balls, you can earn candies. When it is correct, a thumb can appear and you will get a candy that you can eat right away. If it is wrong, nothing will appear. Do you have any questions? If not, you can push the start button. Good luck!” The child was then asked to begin the task. No further instructions were given. If the child requested further clarification or did not start the task, the instructions were repeated. The experimenter remained in the room throughout the task. At the end of all test sessions, all families received 10 euros per session for participating in the study.

The study was approved by the ethical committee of the KU Leuven (Social and Societal Ethics Committee, Faculty Psychology and Educational Sciences, G-2015 01156) and all procedures performed in studies involving human participants were in accordance with the ethical standards of the institutional research committee and with the 1964 declaration of Helsinki and its later amendments.

## Results

### Statistical Analyses

Statistical analyses were performed using SPSS version 24 (IBM, SPSS Software, Armonk, NewYork, USA). Prior to data analysis, we followed standard data cleaning and checking procedures (Tabachnick and Fidell 2014). The distribution of the outcome variables was reviewed and variables with non-normal distributions (high skewness, high kurtosis) and/or extreme values (outliers) were transformed using Square Root transformations. Acquisition and extinction data were analyzed with 2 (group) × 3 (condition) ANOVAs to assess main effects and interactions. In addition, as this was an entirely novel task, strictly exploratory one-way ANOVAs were carried out to allow group comparisons (ADHD versus TD) separately within each condition. Given the sample size and study design, our study had sufficient power (.80) to detect a medium effect size (*f =* .2988) at α = 0.05 (Faul et al. 2007).

Altogether the data from 111 children were included in the analyses. Data from eight children (six ADHD and two TD) were excluded because these children did not meet the study inclusion criteria (IQ < 80: *n* = 4; ADHD diagnosis not confirmed by structured interview: *n* = 3; on medication during assessment: *n* = 1).

### Demographic Characteristics

Based on a (semi-)structured interview, 26 children in the ADHD group met DSM-IV criteria for ADHD-C (combined subtype), 16 children for ADHD-I (inattentive subtype) and 7 children for ADHD-H/I (hyperactive/impulsive subtype).

Demographic characteristics were compared for children with and without ADHD using Chi Square tests and 2 (group) × 3 (condition) ANOVAs (Tables 1 and 2). There was a main effect of group for estimated IQ, *F*(1, 105) = 11.26, *p* = 0.001, *η*^2^_*p*_ = 0.097 (*M*_*ADHD*_ = 97.92; *M*_*TD*_ = 104.79). There was no main effect of condition nor an interaction effect. Despite the significant difference in IQ between children with ADHD and TD children, estimated IQ scores did not correlate with any of the outcome variables and therefore were not used as a covariate in the analyses (Dennis et al. 2009) (see Appendix Table 1).

**Table 1:** Differences in demographic and clinical characteristics in ADHD and TD children

	ADHD	TD	*F/χ*^*2*^	*p*
	M (SD)	M (SD)		
Gender N				
Male	35	33	3.82	.051
Female	14	29		
Age (years)	10.66 (1.02)	10.30 (1.22)	2.79	.098
FSIQ	97.92 (11.18)	104.79 (10.55)	11.26	.001**
Dyscalculia (%)	2.04	0	1.28	.258
Dyslexia (%)	8.16	1.61	2.73	.098
DBDRS (raw score - norm score)				
Attention	16.14 (4.79)	4.15 (3.91)	211.36	<.001***
	14.27 (1.66)	10.58 (1.05)	203.89	<.001***
Hyperactive/impulsive	13.03 (5.73)	3.89 (3.60)	105.22	<.001***
	14.00 (2.05)	10.76 (1.26)	104.74	<.001***
ODD	7.37 (4.96)	2.42 (2.48)	46.99	<.001***
	12.71 (2.28)	10.66 (1.07)	39.31	<.001***
CD	1.20 (1.44)	0.58 (1.25)	5.95	.016*
	11.51 (1.57)	10.97 (1.17)	4.34	.040*

*ADHD* Attention deficit hyperactivity disorder, *TD* Typically developing, *FSIQ* Full scale IQ, *PDISC* Parent diagnostic interview scale, *ADHD-C* Attention deficit hyperactivity disorder combined presentation, *ADHD-I* Attention deficit hyperactivity disorder inattentive presentation, *ADHD-H/I* Attention deficit hyperactivity disorder hyperactive/impulsive presentation, *DBDRS* Disruptive behavior rating scale, *ODD* Oppositional defiant disorder, *CD* Conduct disorder

Diagnosis of dyscalculia and dyslexia is based on parent report

*p < .05, **p < .01, ***p < .001

**Table 2:** Differences in demographic and clinical characteristics between children in the continuous, partial and stretching the ratios condition

		ADHD		TD		*F/χ*^*2*^	*p*
Continuous (n = 37)	N	16		21			
	Age (M/SD)	10.58	1.08	10.47	1.14	0.09	.762
	FSIQ (M/SD)	100.69	11.29	105.19	12.06	1.34	.255
	Gender (M:F)	11:5		12:9		0.52	.471
	Medication (%)	68.75		0			
Partial (n = 37)	N	17		20			
	Age (M/SD)	10.82	1.01	10.24	1.17	2.53	.121
	FSIQ (M/SD)	95.47	11.11	108.15	9.21	14.42	.001**
	Gender (M:F)	12:5		10:10		1.61	.204
	Medication (%)	56.25		0			
Stretching the Ratios (n = 37)	N	16		21			
	Age (M/SD)	10.58	1.03	10.19	1.38	0.91	.346
	FSIQ (M/SD)	97.75	11.20	101.19	9.40	1.03	.317
	Gender (M:F)	12:4		11:10		1.98	.160
	Medication (%)	43.75		0			

*ADHD* Attention deficit hyperactivity disorder, *TD* Typically developing, *FSIQ* Full scale IQ, *M* Male, *F* Female

**p < .01

### Ball Game

We first present the data on pre-acquisition trials, i.e., trials before the children received their first reward, followed by our primary outcome measures for acquisition and extinction. To assess the need to control for the number of pre-acquisition trials, we compared the mean number of trials until the first reward across the ADHD and TD groups using a 2 (group) × 3 (condition) ANOVA. There was a main effect of condition *F*(2, 105) = 3.18, *p* = 0.046, *η*^2^_*p*_ = 0.057. A post-hoc Bonferroni-corrected test showed that participants in the partial condition needed more trials than participants in the stretching the ratios condition to reach their first reward, which is trivial given that their chance to be rewarded for a correct response was only 20% compared to 100% in the stretching the ratios condition (*p* = 0.040). There was no main effect of group nor an interaction effect.

**Fig. 2:**
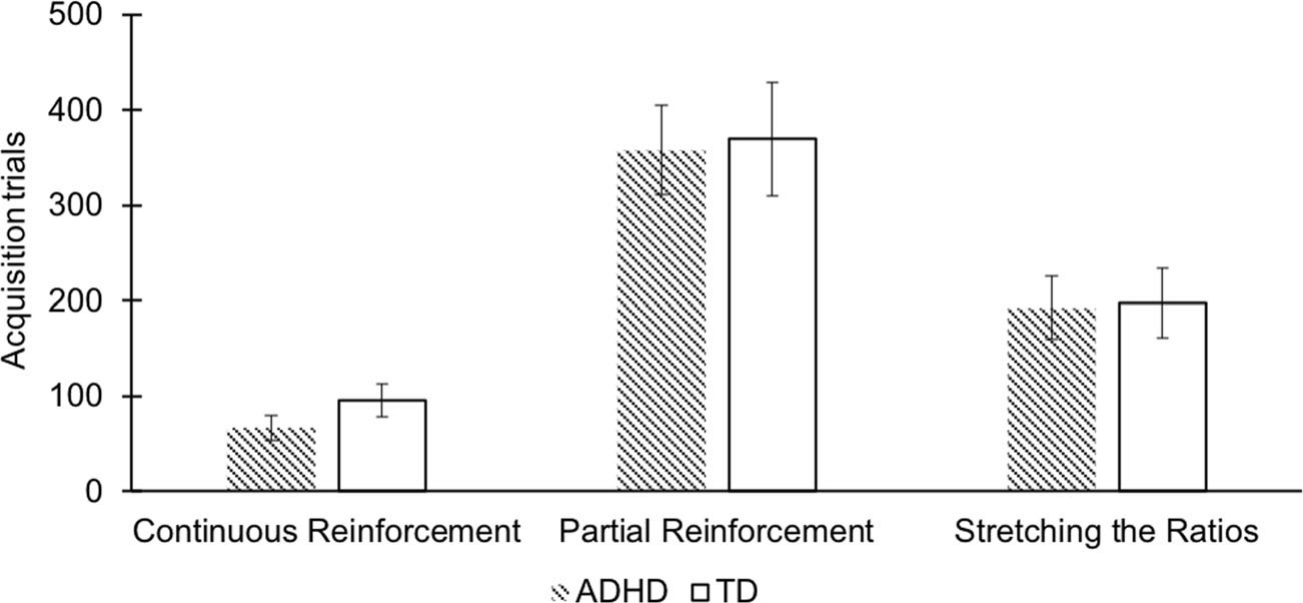
Mean acquisition trials until criterion (20 reinforcements) for children in the ADHD and TD groups across the three reinforcement conditions. The figure shows a main effect of condition but no main effect of group or interaction

A 2 (group) × 3 (condition) ANOVA was conducted to assess main effects and interactions for the number of acquisition trials (number of trials after the first reward was received until 20 rewards were received). In line with our predictions, there was a main effect of condition for the number of acquisition trials, *F*(2, 105) = 32.11, *p* < 0.001, *η*^2^_*p*_ = .380 (Tables 3 and 4).^4^ A Bonferroni-corrected post-hoc test indicated that the number of acquisition trials differed significantly (*p*_*PRF*-*CRF*_ < 0.001, *p*_PRF_-_StR_ < 0.001, *p*_CRF_-_StR_ = 0.001) between all three conditions. The most acquisition trials were recorded in the partial reinforcement condition (*M* = 364.43)^5^ followed by stretching the ratios (*M* = 195.59) and then continuous reinforcement condition (*M* = 81.35). Contrary to our predictions, there were no significant acquisition differences between groups across conditions, i.e., there was no main effect of group (children with ADHD versus TD children) nor a significant interaction (Fig. 2).

**Table 3:** Means, standard deviations and medians of the continuous, partial and stretching the ratios condition before and after square root transformation

Continuous	Total Acquisition Trials		Correct Extinction Trials		Total Extinction Trials	
	ADHD	TD	ADHD	TD	ADHD	TD
M	66.81	92.43	7.31	6.76	51.38	54.62
Mde	52.50	62.00	7.00	6.00	51.00	45.00
SD	53.25	76.16	3.32	3.55	12.71	27.52
M (transformed)	7.65	8.83	2.64	2.53	7.12	7.16
Mde (transformed)	7.24	7.87	2.65	2.45	7.14	6.71
SD (transformed)	2.98	3.89	0.61	0.63	0.88	1.89
Partial	Total Acquisition Trials		Correct Extinction Trials		Total Extinction Trials	
	ADHD	TD	ADHD	TD	ADHD	TD
M	358.35	369.60	18.24	20.75	64.94	80.00
Mde	321.00	369.00	18.00	19.00	66.00	77.00
SD	193.37	263.77	12.94	12.28	18.74	24.07
M (transformed)	18.25	17.80	4.00	4.33	7.97	8.86
Mde (transformed)	17.92	19.07	4.24	4.35	8.12	8.77
SD (transformed)	5.18	7.46	1.54	1.46	1.22	1.25
Stretching the Ratios	Total Acquisition Trials		Correct Extinction Trials		Total Extinction Trials	
	ADHD	TD	ADHD	TD	ADHD	TD
M	192.69	197.81	20.06	18.95	60.44	58.24
Mde	142.00	119.00	22.50	20.00	59.00	59.00
SD	131.20	165.43	11.05	9.99	13.16	14.28
M (transformed)	13.19	12.94	4.28	4.15	7.73	7.57
Mde (transformed)	11.90	10.91	4.74	4.47	7.68	7.68
SD (transformed)	4.47	5.64	1.36	1.36	0.85	0.95

*ADHD* Attention deficit hyperactivity disorder, *TD* Typically developing, *M* Mean, *Mde* Median, *SD *Standard deviation

**Table 4:** Results from 2 (Group: ADHD, TD) x 3 (Condition: PRF, CRF, StR) ANOVAs for transformed acquisition and extinction trials

	Group			Condition			Condition x Group		
	*F*	*p*	*η*^*2*^_*p*_	*F*	*p*	*η*^*2*^_*p*_	*F*	*p*	*η*^*2*^_*p*_
Total Acquisition Trials	0.26	.872	.000	32.11	<.001***	.380	0.27	.767	.005
Correct Extinction Trials	0.01	.905	.000	20.95	<.001***	.285	0.41	.662	.008
Total Extinction Trials	1.15	.286	.011	9.61	<.001***	.155	1.79	.171	.033

*ADHD* Attention deficit hyperactivity disorder, *TD* Typically developing, *CRF* Continuous reinforcement, *PRF* Partial reinforcement, *StR* Stretching the ratios

***p < .001

During extinction, a main effect of condition was found for the number of correct responses, *F*(2, 105) = 20.95, *p* < 0.001, *η*^2^_*p*_ = .285 (Tables 3 and 4). Post-hoc Bonferroni-corrected tests indicated that children in the partial and stretching the ratios condition did not differ in the number of correct trials during extinction, but children in the continuous condition (*M* = 7.00) made significantly fewer correct responses than those in the partial (*M* = 19.59, *p* < 0.001) and the stretching the ratios conditions (*M* = 19.43, *p* < 0.001). A main effect of condition was also observed for the total number of extinction trials, *F*(2, 105) = 9.61, *p* < 0.001, *η*^2^_*p*_ = .155 (Tables 3 and 4). Post-hoc comparisons indicated that children in the partial condition (*M* = 73.08) emitted significantly more responses than the children in the stretching the ratios condition (*M* = 59.19, *p* = 0.020) and those in the continuous condition (*M* = 53.22, *p* < 0.001). The two groups performed similarly across all three conditions, for both extinction measures, resulting in the absence of main effects for group or group by condition interactions.

**Fig. 3:**
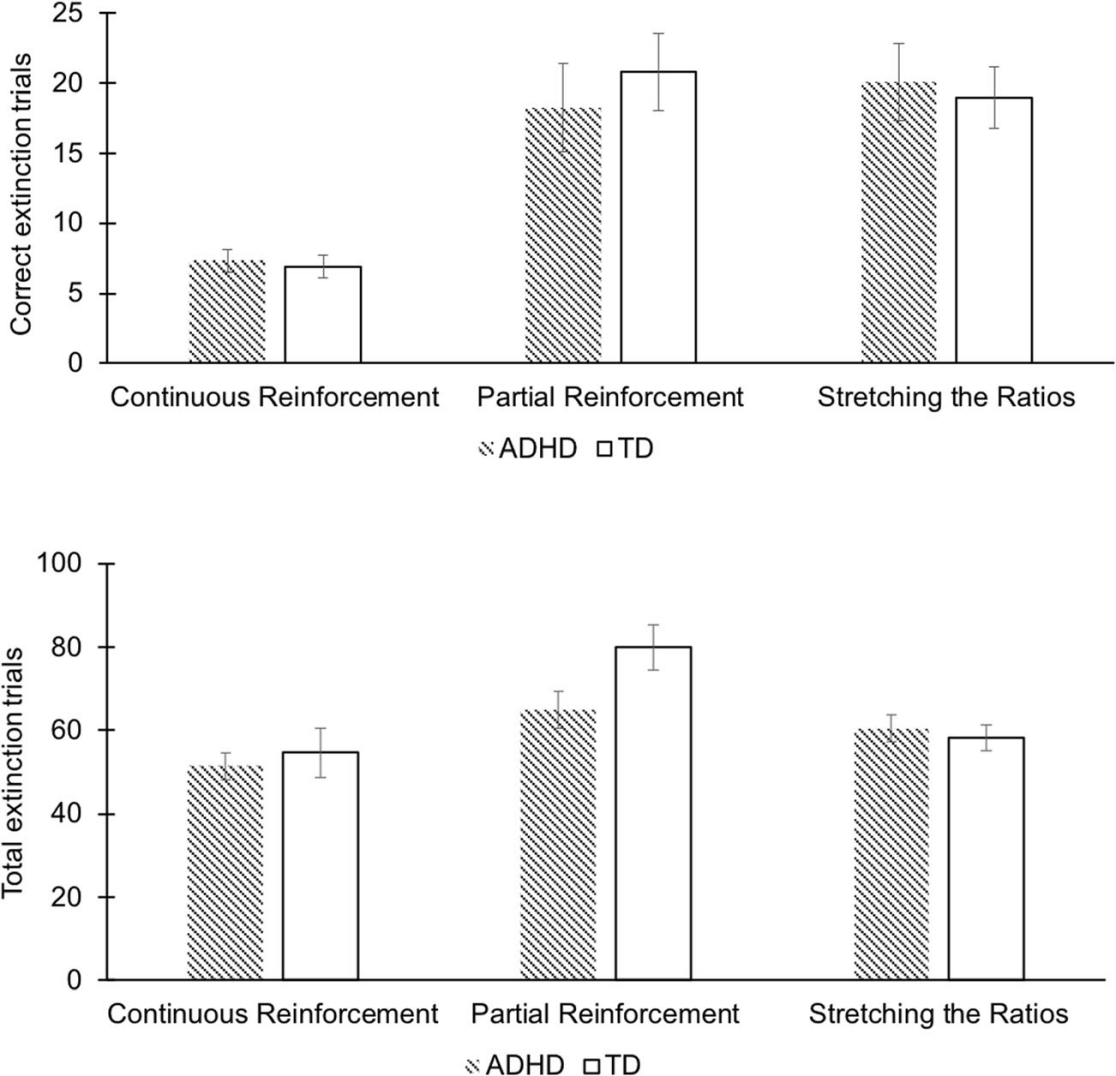
Mean number of correct responses during extinction (top panel) and mean total number of responses during extinction (bottom panel) across the three reinforcement conditions for children in the ADHD and TD groups. The figure shows main effects of condition but no main effects of group or interactions

Despite the absence of any group effects in the omnibus ANOVAs, we performed a series of strictly exploratory one-way ANOVAs on the acquisition and extinction data. These analyses yielded no significant effects in acquisition. There was a significant difference between TD children and children with ADHD in the total number of extinction trials in the partial reinforcement condition, *F*_*PRF*_ (1, 35) = 4.78, *p* = 0.036, *ω* = .304 (Table 5). In this condition, the children with ADHD appeared to make fewer responses during extinction than the TD children. However, the effect did not meet a Bonferroni-corrected threshold (*p* < 0.025). No group differences were found for the total number of extinction responses in the continuous and stretching the ratios acquisition conditions (Fig. 3).

**Table 5:** Exploratory analysis of Groups (ADHD vs TD) through ANOVAs on transformed acquisition and extinction trials

	CRF		PRF		StR	
	F	*p*	F	*p*	F	*p*
Total Acquisition Trials	1.03	.318	0.05	.834	0.02	.887
Correct Extinction Trials	0.29	.592	0.44	.512	0.09	.771
Total Extinction Trials	0.01	.940	4.78	.036*	0.27	.610

*ADHD* Attention deficit hyperactivity disorder, *TD* Typically developing, *CRF* Continuous reinforcement, *PRF* Partial reinforcement, *StR* Stretching the ratios

*p < .05

## Discussion

The present study examined instrumental reinforcement learning (acquisition) and extinction under conditions of partial and continuous reinforcement in children with ADHD and TD children, using a newly-developed task, closely modeled on procedures used in operant learning experiments in animals (Segers et al. 2018; Sonuga-Barke 2003). The new task was successful in that both ADHD and TD children were able to complete the task under all three reinforcement conditions and that our reinforcement manipulations were effective in modulating acquisition and extinction performance.

As predicted, children across groups showed faster acquisition under continuous than under partial reinforcement. Learning under a stretching-the-ratios schedule was faster than under partial reinforcement across both groups of children as well. In contrast to the findings from a small number of earlier experimental studies on learning under partial versus continuous reinforcement (Douglas and Parry 1994; Freibergs and Douglas 1969; Parry and Douglas 1983), we did not see a deficit in acquisition under partial reinforcement in children with ADHD. Their performance in acquisition under conditions of partial reinforcement, as under other reinforcement conditions, was similar to that seen in TD children. During extinction, as expected, behavior acquired under conditions of partial reinforcement was more persistent than behavior acquired under continuous reinforcement, for those with and without ADHD alike, providing evidence of a Partial Reinforcement Extinction Effect in both groups.

Whereas both Sagvolden’s (2005) Dynamic Developmental theory and Tripp and Wickens' (2008) Dopamine Transfer Deficit theory of ADHD predict poorer acquisition under conditions of partial reinforcement in children with ADHD due to hypofunctioning of the dopamine system, the current findings do not support this prediction. Furthermore, the results of the current study are not consistent with the predictions made by either theory regarding sensitivity to extinction. In the current study, children with ADHD showed neither slower (Sagvolden et al. 2005) nor faster (Tripp and Wickens 2008) extinction than TD children.

With the aim of addressing possible Partial Reinforcement Extinction deficits in children with ADHD, learning under increasing ratio strain (stretching the ratios) was also explored. Compared to learning under partial reinforcement, stretching the ratios was effective in increasing the speed of acquisition (i.e., the number of trials required to obtain 20 rewards) and in establishing behavioral persistence (PREE) during extinction in both TD children and those with ADHD, and the effectiveness of stretching the ratios appeared again mostly similar for both groups.

Further exploratory analyses did uncover a potential difference in extinction performance between children with ADHD and TD children, although this finding did not survive Bonferroni correction. When reinforcement was no longer available during the extinction phase, it should be adaptive to engage in exploratory behavior, i.e., by examining other options (balls) that might lead to reinforcement. There seemed to be a trend towards less such exploratory behavior in children with ADHD; those who acquired the correct response under partial reinforcement conditions were less likely to engage in other behaviors to obtain rewards during extinction. It may be that children with ADHD, compared to TD children, have a greater difficulty adapting their behavior to a changing environment (e.g., when previously reinforced behavior is no longer or infrequently followed by a reinforcer) (Alsop et al. 2016; Barkley 1997; Furukawa et al. 2018). This tentative difference, if confirmed in future research, might reflect a reduction in motivation in children with ADHD to find reinforcement, especially when an unpredictable reward schedule during acquisition is followed by an unannounced contingency change (Alsop et al. 2016). However, this is the first study with this task and the significant between-group difference, which resulted from a post-hoc data analysis rather than a pre-defined hypothesis, did not survive Bonferroni correction. Moreover, in the absence of validation with daily life behavior, we cannot know for certain that this altered exploratory behavior in children with ADHD, if genuine, represents a deficit. Any interpretation of this findings should therefore be made with considerable caution.

Tentatively, it may be attributed to frustration from non-reward under partial reinforcement (Amsel, 1994). In the only study on this topic in children with ADHD, it was observed that compared to TD children, they exhibited more frustration during acquisition under partial reinforcement and extinction conditions (Wigal et al. 1998). Perhaps then, a tendency towards reduced exploratory behavior in children with ADHD during extinction after partial reinforcement learning, if corroborated in future research, might stem from higher levels of frustration generated in a partial reinforcement condition which interferes exploratory behavior. Future research may want to integrate measures of frustration in reinforcement learning tasks under partial and continuous reinforcement to directly test this hypothesis.

A relevant extension for future research might also be to explore the effects of primary versus secondary reinforcers on partial reinforcement learning. In the current task, using both primary (candy) and secondary (thumbs up) reinforcers, we did *not* find differences in acquisition between children with ADHD and TD children; this allowed unambiguous interpretation of extinction performance in children with ADHD, unconfounded by any performance differences between the ADHD and TD groups originating in acquisition. However, the strong reinforcement salience/value generated by the inclusion of both primary and secondary reward may have been sufficiently powerful to obscure subtle acquisition deficits in reinforcement learning under conditions of partial reinforcement, by amplifying the contingency between response and reward.

Finally, the absence of group differences in acquisition and extinction might also be due to the nature of the task itself. While our results clearly show that children with ADHD are able to learn the task, its simplicity might have contributed to the equal level of performance across groups. Follow-up research could explore a more challenging task (without confounding reinforcement learning processes), and the effects of partial reinforcement learning in ADHD vs TD when only secondary and thus less salient reinforcers (thumbs up) are used.

Future studies should also assess the potential role of working memory in basic reinforcement-learning deficits, as impairments in executive functioning are often reported in children with ADHD (Dovis et al. 2012; Martinussen et al. 2005) and a potential load of reinforcement learning tasks on working memory or related cognitive abilities might have caused group differences in earlier studies (Freibergs and Douglas 1969; Segers et al. 2018). Our task was designed to be relatively simple but considering that in our task, initially, participants need to remember the color they have pressed, we cannot be sure that working memory did not contribute to performance. However, exploratory analyses with a subset of the current sample (part of another study) showed there were no significant correlations between working memory performance (Corsi Block Tapping Task) and our outcome variables (see Appendix Table 1).

In interpreting the current findings, it is important to acknowledge the limitations of our study. The between-subjects design reduced sample sizes and therefore the power to detect differences between reinforcement conditions. Replication with a larger sample would allow us to explore in more detail the role of neuropsychological and pathological heterogeneity within ADHD. The experimental task was specifically developed for the study and has yet to be replicated and validated in relation to daily life behavior. Our results do indicate that it worked as designed, i.e., there were differences in acquisition across the three conditions and a clear Partial Reinforcement Extinction Effect was observed in ADHD and TD children alike.

In designing the study, our focus was on the influence of differences in relative reinforcement rate, not absolute amount of reinforcement. Therefore, across conditions children received the same number of reinforcers. This necessarily implied differences in the number of trials between conditions (children in the partial condition had to perform more trials to reach the reinforcement criterion than children in the continuous condition), which could have influenced the results. While this feature is inherent to the design of any study comparing continuous to partial reinforcement, it has to be taken into account when interpreting the results. The design of the study does, however, allow us to directly compare children in the ADHD group to those in the TD group within each condition. Another inherent feature of the design is the combined manipulations of reinforcement frequency and timing in the stretching-the-ratios condition. Note that this is considered a basic feature of a stretching-the-ratios manipulation that is known to enhance the power of this procedure (Bouton and Sunsay 2001; Sangha et al. 2003; Skinner 1968).

Task completion time was also not recorded and therefore cannot be used to rule out effects of fatigue on performance on the task, although it was observed that children readily kept responding until 20 reinforcements were obtained in all conditions. Another task-related limitation is the timing of testing. The current task was always presented at the end of the first or last test session for a child, without counterbalancing.

Other limitations of the study are related to the sample. As the ADHD group included children with ODD, it is possible that the reduction in exploratory behavior in extinction after partial reinforcement, if genuine, may be due to comorbid ODD rather than ADHD symptoms. Motivational deficits are also found in children with ODD (Angold et al. 1999; Matthys et al. 2012). In the absence of a pure ODD group this is difficult to disentangle. However, within the ADHD sample ODD symptoms (as rated by the DBDRS) did not correlate significantly with the total number of extinction trials. Further, we did not succeed in collecting teacher data to confirm the ADHD diagnosis for all participants. Missing teacher data were the result of technical constraints (i.e., no response, children changing teachers, absence of contact information, data collection continuing through the summer vacation when teachers were not available). While we acknowledge that this is a limitation, most of the children who entered the study had been previously assessed and diagnosed through the KU Leuven university hospital, by means of multi-method, multi-informant assessments. Conversely, the children in the TD group were not subjected to (semi-)structured clinical interviews to confirm the absence of an ADHD diagnosis. However, they all had scores within the normal range on the DBDRS.

The current study also has a number of important strengths. Key amongst these was the use of a very basic and simple learning task, limiting the possible role of confounding factors such as cognitive abilities. In previous studies more complex tasks were used, measuring such things as delayed reaction time or spellings mistakes, potentially confounding measurement of reinforcement learning with working memory, intelligence or executive functions (Barber et al. 1996; Frank et al. 2007; Segers et al. 2018). This is especially important as numerous studies also report working memory deficits in those with ADHD (Dovis et al. 2013; Martinussen et al. 2005).

A second strength of the present study relates to the fact that we took into account both the number of correct trials and the total number of trials during extinction as two distinct measures of extinction performance. By doing this, we are able to delineate extinction effects more clearly than in previous studies. These previous studies often considered only the total number of trials per minute in extinction as the outcome variable (e.g., Saini et al. 2017) or even drew conclusions about the PREE on the basis of acquisition data only (Parry and Douglas 1983). Finally, most previous studies on reinforcement learning in ADHD included participants diagnosed with less stringent diagnostic inclusion criteria (Freibergs and Douglas 1969; Morgan et al. 1996; Parry and Douglas 1983), whereas all the participants in our study met DSM-IV criteria for ADHD.

The results of the current study have clear and important clinical implications. Reinforcement contingencies are a cornerstone of Behavior Therapy for ADHD (Lee et al. 2012). Although a backbone of BT, the specific response to reinforcement contingencies of children with ADHD has received little empirical attention. This is remarkable given that reinforcement deficits have been hypothesized to be central to the disorder (Luman et al. 2005; Sagvolden et al. 2005; Sonuga-Barke 2002). Our results show that the use of a sufficiently potent reinforcer that is presented immediately after the target response, even on an intermittent schedule only, establishes learned behavior at the same rate in children with ADHD as in their typically developing peers.

The current data also suggest that, similarly to typically developing children, stretching the ratios may be useful in establishing behavioral persistence in children with ADHD. In behavior therapy for children with ADHD, parents or teachers are typically instructed to provide continuous reinforcement to install adaptive behavior in a child. The downside of this is that behavior that is learned under continuous reinforcement will extinguish faster when reinforcement is removed than is the case for behavior learned under partial reinforcement. Our results indicate that children with ADHD acquire new adaptive behavior faster when reinforcement progresses gradually from a continuous to a partial scheme (stretching the ratios), compared to partial reinforcement throughout. Yet, when reinforcement is subsequently removed, like TD children, children with ADHD show the same behavioral persistence as observed after partial reinforcement. Moreover, to the extent that there is a tendency towards reduced behavioral exploration under extinction in children with ADHD after partial reinforcement, this tendency seems absent after gradual ratio stretching.

Translating this to clinical practice, ratio stretching could be routinely integrated in behavioral treatment for children with ADHD, by instructing caregivers to provide high rates of reinforcement early on in the acquisition of desired behavior to then gradually proceed to lower levels of reinforcement. Such schedule might better prepare children to deal with later discontinuation of reinforcement while at the same time preventing the frustration and the possibly altered exploratory behavior associated with partial reinforcement ab initio. Although some BT programs have stretching of reinforcement ratios integrated (Van Den Hoofdakker et al. 2007), to our knowledge most BT programs for ADHD do not explicitly use this principle (Daley et al. 2018; Kaminski et al. 2008). Yet our results suggest that under ratio stretching, adaptive behavior will be learned faster while retaining persistence when reinforcement is discontinued.

## Electronic supplementary material

ESM 1 (DOCX 24 kb)

## Footnotes

1 50 children

2 5 children

3 With the exception of 3 children for whom only a full IQ score was available.

4 Analyses were carried out on the transformed data

5 Untransformed means are included in Table 3 for ease of interpretation
