# Supplementary material for: Reinforcement Contingency Learning in Children with ADHD: Back to the Basics of Behavior Therapy
Source: J Abnorm Child Psychol. 2019 Jul 11;47(12):1889–902. doi: 10.1007/s10802-019-00572-z (PMC6842356; doi:10.1007/s10802-019-00572-z)
Supplement: Supplementary file 1 — (DOCX 24 kb) [file 10802_2019_572_MOESM1_ESM.docx]

| Appendix Table 1 | | | | | |
| --- | --- | --- | --- | --- | --- |
| *Partial and Bivariate Correlations between the Corsi Block Tapping Task, IQ and the outcome variables on CRF, PRF and StR.* | | | | | |
| CRF | 1 | 2 | 3 | 4 | 5 |
| 1. IQ^2^ | 1 |  |  |  |  |
| 1. CBTT-BW^1^ | -.003 | 1 |  |  |  |
| 1. Acquisition^2^ | -.011 | .151 | 1 |  |  |
| 1. Extinction (Correct)^2^ | .011 | .026 | .343* | 1 |  |
| 1. Extinction (Total)^2^ | .135 | .159 | .541** | .705*** | 1 |
| PRF |  |  |  |  |  |
| 1. IQ^2^ | 1 |  |  |  |  |
| 1. CBTT-BW^1^ | .361 | 1 |  |  |  |
| 1. Acquisition^2^ | .032 | .283 | 1 |  |  |
| 1. Extinction (Correct)^2^ | .156 | -.204 | -.760*** | 1 |  |
| 1. Extinction (Total)^2^ | .317 | .121 | .503** | -.366* | 1 |
| StR |  |  |  |  |  |
| 1. IQ^2^ | 1 |  |  |  |  |
| 1. CBTT-BW^1^ | .144 | 1 |  |  |  |
| 1. Acquisition^2^ | -.077 | -.248 | 1 |  |  |
| 1. Extinction (Correct)^2^ | .006 | -.410* | -.480* | 1 |  |
| 1. Extinction (Total)^2^ | -.265 | -.256 | .242 | .297 | 1 |
| * correlation is significant at the .05 level (2-tailed)  ** correlation is significant at the .01 level (2-tailed)  *** correlation is significant at the .001 level (2-tailed)  ^1^ Partial correlation (controlled for age)  ^2^ Bivariate correlation | | | | | |
